# Expression of concern: necrotizing fasciitis, literature review of contemporary strategies for diagnosing and management with three case reports: torso, abdominal wall, upper and lower limbs

**DOI:** 10.1186/1749-7922-7-33

**Published:** 2012-10-18

**Authors:** Fausto Catena and Frederick Moore, Editors-in-Chief, World Journal of Emergency Surgery

## Comment on

Zdravko Roje, Željka Roje, Dario Matić, Davor Librenjak, Stjepan Dokuzović and Josip Varvodić. Necrotizing fasciitis: literature review of contemporary strategies for diagnosing and management with three case reports: torso, abdominal wall, upper and lower limbs. *World Journal of Emergency Surgery* 2011 **6**:46. doi: http://10.1186/1749-7922-6-46. URL http://www.wjes.org/content/6/1/46.

The Editors-in-Chief would like to alert readers that the accuracy of details described in Case III (56 year old male) of this article [1] have been questioned by a reader. Unfortunately, for legal reasons, the authors’ institution has not been able to confirm or refute whether the events reported about the case are accurate. Consequently, readers are advised to interpret the details of Case III with caution.
